# Nutrient timing habits of Division I NCAA athletes

**DOI:** 10.1186/1550-2783-12-S1-P33

**Published:** 2015-09-21

**Authors:** M G Nystrom, AR Jagim, M Greenwood, J M Oliver, MT Jones

**Affiliations:** 1 Kinesiology Department, Texas Christian University, Fort Worth, TX, 76129, USA; 2 Exercise & Sport Science Department, University of Wisconsin - La Crosse, La Crosse, WI, 54603, USA; 3 Health and Kinesiology Department, Texas A&M University, College Station, TX 77840, USA; 4 Division of Health and Human Performance, George Mason University, Fairfax, VA, 22030, USA

## Background

It has been suggested that nutrient timing strategies may augment training adaptations in active populations. However, collegiate athletes are often restricted by practice schedules, class times and training sessions and, as a result, may not follow recommendations on optimal feeding strategies. Therefore, a survey questionnaire, which examined the nutrient timing habits of athletes, was designed and administered at selected Division I Institutions within the United States.

## Methods

A total of 481 (240 women, 241 men) NCAA Division I athletes representing eleven intercollegiate sports from three universities in three athletic conferences (i.e., Atlantic 10, Atlantic Coast Conference, Conference USA) volunteered to participate as subjects. There were 18 multiple choice questions that addressed nutrient timing habits. The surveys were administered to all athletes during a scheduled training time.

## Results

When asked about breakfast habits 2% (9/398) reported eating breakfast ≤ once per week, while 51% (204/398) reported consuming breakfast 7 days per week. 79% of all athletes reported feeling hungry prior to training, practice or competition. However, 77% of all athletes surveyed reported that it was "*easy*" to eat 1-2 hours prior to competition. A summary of the amount of time prior to practice, training or competition that athletes consumed a full meal is presented in Table 1. When asked if they "snacked" during practice, 24% of men and 23% women responded positively. Only 51% percent of all athletes reported that their athletic department provides post workout or game day nutrition. When asked how soon after practice, training and competition they consumed a full meal, 2% responded 15 min, 15% responded 30 min, 22% responded 45 min, 44% responded 1 hr and 17% responded 2 hr. In regard to nutritional periodization, 43% of men reported that they consume the same number of calories during off-season and in-season with 36% reporting that they were unsure. Similarly, 22% of women reported that they eat the same amount of calories during off-season and in-season with 38% reporting they were unsure.

**Table 1:** Summary of meal consumption prior to training or competition.

Hours Before	Men	Women	Totals
6 hrs.	5	7	12

5 hrs.	2	2	4

4 hrs.	16	22	38

3 hrs.	62	57	119

2 hrs.	91	86	177

1 hr.	24	28	50

Totals	200	200	400

Total % Responses	100%	97%	99%

## Conclusions

It appears as though most athletes consumed breakfast regularly throughout the week. In addition the results suggest that athletes are consuming a meal or snack after training and competition despite the fact that only 51% of athletes reported their athletic departments provide post-workout nutrition. However, the majority of athletes also reported feeling hungry prior to training. It is suggested that more proactive strategies may need to be employed to optimize training adaptations.

